# Encapsulation, Shelf Life, and Virulence of *Batkoa* sp. Against *Dalbulus maidis*

**DOI:** 10.3390/jof10120814

**Published:** 2024-11-23

**Authors:** Daniela Milanez Silva, Natasha Sant’ Anna Iwanicki, Linda Claire Muskat, Anant V. Patel, Italo Delalibera Júnior

**Affiliations:** 1 Department of Entomology and Acarology, “Luiz de Queiróz” College of Agriculture, University of São Paulo (ESALQ/USP), Piracicaba 13418-900, Brazil; iwanicka1@gmail.com (N.S.A.I.); delalibera@usp.br (I.D.J.); 2 Department of Crop Protection, Hochschule Geisenheim University, Von-Lade-Straße 1, 65366 Geisenheim, Germany; linda.muskat@hs-gm.de; 3 Fermentation and Formulation of Biologicals and Chemicals, Faculty of Engineering and Mathematics, Bielefeld University of Applied Sciences, Interaktion 1, 33619 Bielefeld, Germany; anant.patel@hsbi.de

**Keywords:** Entomophthorales, biological control, formulation, entomopathogenic fungus, fluidized bed drying

## Abstract

*Batkoa* is a genus of entomophthoralean fungi often associated with insect epizootics, particularly in phytophagous hemipterans. Encapsulation has become a promising strategy for improving the shelf life and sporulation of these fungi post-application. This study aims to (i) compare the virulence of the submerged propagules and primary conidia of *Batkoa* sp. ESALQ1199 against *Dalbulus maidis*; (ii) formulate submerged propagules in calcium alginate beads with co-formulants; (iii) assess the colony-forming units and sporulation of encapsulated beads dried with different kaolin concentrations (0%, 2%, 4%, 8% and 10%); (iv) determine the shelf life of dried bead formulations containing 10% kaolin, comparing washed and unwashed beads treated with a 4% sucrose solution; and (v) assess the sporulation capacity of beads with 10% kaolin, washed and unwashed with 4% sucrose solution, over time under humid conditions. Our results demonstrated that primary conidia and submerged propagules effectively killed 82.4% and 57.8% of adult corn leafhoppers, respectively. Co-formulants maintained viability above 80% in dried propagules, while control samples dropped to 45%, indicating the sensitivity of submerged propagules to the drying process. Encapsulated *Batkoa* sp. retained the same concentration of viable propagules per bead and the number of conidia produced (sporulation) for 30 days at 28 °C. The sporulation of fresh beads increased during the incubation period, plateauing after 27 days. This suggests that *Batkoa* sp. beads can produce primary conidia under humid field conditions, serving as a potential inoculum source for new infections.

## 1. Introduction

The genus *Batkoa* (Humber) is one of the few entomophthoralean fungi with a broad host range frequently associated with insect epizootics, primarily within the order Hemiptera [1]. The generic name *Batkoa* was first proposed in 1989 by Richard Humber [2], although the taxonomy of the *Batkoa* group has undergone significant revisions in recent years, highlighted by the creation of the family Batkoaceae in 2022 [1]. This taxonomic update was implemented to consolidate species of *Batkoa* and to reclassify some *Conidiobolus* species to the genus *Batkoa*, as exemplified by the renaming of *Conidiobolus obscura* to *Batkoa obscura* (Hall & Dunn) Gryganskyi, comb. nov. The newly proposed Batkoaceae arises basal to the family Entomophthoraceae, with eleven species formally described [3], occurring across the Americas, Europe, Oceania, and Asia [4,5].

In Brazil, the species *Batkoa major* (Thaxt.) Humber and *Batkoa apiculata* (Thaxt.) Humber have been identified as infecting economically important hemipteran pests in agricultural fields, such as *Mahanarva fimbriolata* [6,7], *Deois flavopicta*, *Zulia entreriana* [4,8], and more recently the corn leafhopper *Dalbulus maidis* [9]. The specificity of certain *Batkoa* species to hemipterans offers an additional tool for controlling these pests, as their control rates during epizootic events surpass the average achieved by other entomopathogenic fungi, particularly those from the Hypocreales order, such as *Metarhizium anisopliae* and *Beauveria bassiana* [7,8,10]. Alves [7] recommended the control of *M. fimbriolata* in Brazilian sugarcane and pasture by introducing cadavers infected with *Batkoa*. Introducing fungus-infected cadavers resulted in approximately 90% insect control in sugarcane fields. Despite being primarily associated with *M. fimbriolata* in Brazil, the fungus *Batkoa* also infects the corn leafhopper *D. maidis*, a primary maize pest with greater economic significance than *M. fimbriolata*.

The corn leafhopper *D. maidis* poses a significant threat to maize crops in Latin America. This insect not only inflicts direct harm on corn plants through its feeding behavior and injection of toxic saliva, but also acts as a vector for several destructive plant pathogens, including maize bushy stunt phytoplasma (MBSP), corn stunt spiroplasma (CSS), and maize rayado fino virus (MRFV) [11,12]. Conventional management approaches for *D. maidis* have relied on chemical insecticides. However, the excessive use and misapplication of these chemicals have spurred the development of resistance [13], highlighting the urgent need for more sustainable control methods. Entomopathogenic fungi have emerged as an effective alternative in the integrated pest management (IPM) of *D. maidis*. Hypocrealean species such as *Cordyceps javanica*, *Beauveria bassiana*, *Metarhizium anisopliae*, and *M. robertsii* have demonstrated efficacy against this pest [11,12,14,15]. While numerous products based on Hypocrealean strains are commercially available as biopesticides, none are based on entomophthoralean fungi. The main reasons for this are the difficulty in establishing a mass production system and the scarcity of formulation studies to preserve the fungus’s viability and efficacy under field conditions.

Formulation represents a critical challenge in the development of bioproducts. Reducing metabolism through drying processes is crucial for fungi to attain low water activity levels, to ensure shelf life, and to prevent undesired contamination [16,17]. During drying, co-formulants such as osmoprotectants, thermoprotectants, and antioxidants can be integrated, mainly when dealing with propagules cultivated in a liquid medium. These additives can form part of the final formulation, which may be further supplemented with additional co-formulants such as inert substances, UVB protectors, and others [15,18,19]. Most commercial formulations of Hypocrealean fungi are wettable powders, oil suspensions, and dispersible granules [20,21]. Conversely, no such formulations are available containing entomophthoralean species as the active agent, and this area remains relatively unexplored.

The encapsulation of entomophthoralean fungi has recently gained prominence as a formulation strategy [22]. This technique offers benefits such as improved shelf life, compatibility, and enhanced sporulation post-application in agricultural fields. Calcium alginate stands out as a particularly compatible carrier for entomophthoralean fungi [23,24], exhibiting promising results in shielding the microbial agent from environmental stressors like drought and UV radiation, which commonly affect the viability of microorganism-based products. These advantages stem from the judicious selection of carriers and fillers, creating an optimal microenvironment conducive to fungal survival [25]. However, the beads formed through the calcium alginate encapsulation method require drying. The fluidized bed dryer is a standard tool for drying granular or particulate solids; it utilizes hot air flow as the drying medium at elevated or near-atmospheric pressure [26]. During drying, the inlet temperature determines the water evaporation rate, with higher temperatures leading to a reduced drying period [27,28]. One advantage of this method is its rapid drying capability, making it suitable for industrial-scale applications. However, a notable drawback and primary challenge is the thermal, osmotic, and oxidative stress experienced by the microorganismal propagules. To address this issue, specific co-formulants should be incorporated during encapsulation for effective drying in the fluidized bed. 

## 2. Materials and Methods

### 2.1. Isolate Selection and Fungus Cultivation

The *Batkoa* sp. isolate ESALQ1199 used in this study originated from an infected *M. fimbriolata* specimen, which was collected in Amélia Rodriguez City, Bahia state, Brazil. The strain is deposited in the Entomopathogenic Microorganisms Collection “Prof. Sérgio Batista Alves” at the Laboratory of Pathology and Microbial Control of Insects, Department of Entomology and Acarology of the University of São Paulo (ESALQ-USP). In this paper, the fungus will be referred to as *Batkoa*. The fungus was cultivated on a solid medium comprising 4.0 g/L potato extract, 20.0 g/L dextrose, and 15.0 g/L agar (potato dextrose agar, PDA), poured in Petri dishes (diameter 90 mm), and incubated at 28 °C under a 12 h photoperiod for ten days. After sporulation, pieces of primary conidia and culture medium were removed from the Petri dishes and transferred to cryovials containing a 10% glycerol solution. The resulting stock solution was preserved in an ultra-freezer at −80 °C.

#### Isolate Identification

For molecular identification, the 18S and 28S rRNA regions were sequenced following the protocol described by Gryganskyi et al. [1]. Notably, our isolate did not cluster with any known species within the genus *Batkoa*, suggesting it represents a novel species. However, due to the unresolved phylogenetic relationships within this group, further taxonomic identification has not been pursued at this stage. Therefore, a morphological description of the observed fungal structures of Batkoa sp. ESALQ1199 is given: Primary conidia are globose, measuring 10–15 µm, and are characterized by a short, overpapillar ring-like neck. These conidia exhibit semi-globose papillae that protrude abruptly from the conidial outline, giving them a distinctive appearance. The secondary conidia share a similar morphology but are gradually more diminutive, maintaining the characteristic papillae and overall structure. The fungus demonstrates robust growth and efficient sporulation on artificial media, particularly on potato dextrose agar (PDA), forming a dense, filamentous, cream-white primary mycelium. When cultivated in submerged media, the fungus produces propagules that strongly resemble the secondary conidia, though they are typically smaller and retain the papillae. Additionally, during fermentation, the fungus also produces segmented hyphal bodies.

### 2.2. Submerged Cultivation

The fungus was cultivated in a liquid medium, as described by Jackson & Jaronski [16]. The carbon source used was anhydrous glucose (81 g L^−1^, 40% total carbon, Carl Roth GmbH + Co., Karlsruhe, Germany, batch No. 6887-3), supplemented with yeast extract (10% total nitrogen, Carl Roth GmbH + Co., Karlsruhe, Germany, batch No. 2363-3) as the nitrogen source (9 g L^−1^). All media contained salts, comprising KH_2_PO_4_ (4.0 g L^−1^), CaCl_2_·2H_2_O, (0.8 g L^−1^), MgSO_4_·7H_2_O (0.6 g L^−1^), and FeSO_4_·7H_2_O (0.1 g L^−1^). The pH of the liquid medium was adjusted to 5.5.

Conidial suspensions were obtained from 10-day-old sporulated cultures grown on PDA plates by harvesting conidia with 10 mL of 0.04% aqueous solution of sorbitan monooleate (Tween^®^ 80, Sigma-Aldrich, St. Louis, MO, USA). Subsequently, the suspension was serially diluted, and conidia concentration was determined using a Neubauer chamber (hemocytometer) at 400× magnification. The fungal inoculum was standardized with aliquots of 5 mL containing 5 × 10^6^ primary conidia mL^−1^, which were then added to baffled Erlenmeyer flasks (250 mL) (Duran, LifeSciences^®^, Wertheim, Germany, Retrace Code: 10133155) filled with 45 mL of liquid medium, resulting in a total volume of 50 mL per flask. Liquid cultures were inoculated and incubated under natural light on a rotary shaker (KS 4000 IC Control, IKA^®^, Staufen, Germany) set at 300 revolutions per minute (rpm) and maintained at 28 ± 2 °C for a 6-day incubation period. All culture flasks were manually shaken daily to prevent mycelial growth on the flask walls. 

Throughout the incubation period, samples were periodically collected to assess culture morphology, and the presence of any contamination was monitored.

### 2.3. Bioassay with Dalbulus maidis

The virulence of the submerged propagules and primary conidia of *Batkoa* was evaluated against *D. maidis*. Corn seedlings (Paraguaçu variety) were planted in 500 mL medium-sized cups filled with the substrate Tropstrato HT Hortaliças, Vida Verde (Grupo Provaso^®^, Mogi Mirim, Brazil). Pots comprised two to three maize plants 30–40 cm in height, were confined inside a cylindrical cage made of an acetate sheet 30–40 cm tall, and were topped with a screen cover made of nylon voile fabric fastened with a rubber band [14]. Ten to fifteen unsexed adults (8–10 days post-emergence) obtained from a colony kept at the Insect Vectors of Plant Pathogens Laboratory at the University of São Paulo, Brazil, were transferred to each cage 24 h before spraying for acclimatization. Submerged propagules and primary conidia were obtained from Petri dishes and liquid culture, as described in Section 2.1 and Section 2.2, respectively. The suspensions applied via spraying contained 1 × 10^7^ primary conidia or submerged propagules in mL^−1^ and 0.01% Silwet^®^ L-77 surfactant (OSi Specialties, Inc., Danbury, CT, USA). The control treatment consisted of sterile distilled water with 0.01% Silwet^®^ L-77 surfactant. An airbrush was used to apply 2 mL of each treatment directly to the insects. Mortality was evaluated daily for ten days post-spraying. The cadavers were transferred to a humid chamber and kept at 27 °C with a 12 h photoperiod to promote sporulation. Each treatment included seven replicates (cages). The seedlings were watered daily with distilled water as needed and maintained in a room at 26–28 °C with a 12 h photoperiod. The bioassay was repeated twice using different insect cohorts and fungal preparations.

### 2.4. Formulation of Batkoa *sp.* Submerged Propagules

Preliminary studies were conducted to evaluate the compatibility of submerged propagules of the fungus *Batkoa* sp. ESALQ1199, mixed individually with various co-formulants, including osmoprotectants and inert substances as filling agents. Based on these studies, kaolin was selected as the inert substance and filling agent, while sucrose and glycerol were identified as the most effective osmoprotectants. Based on these findings, we conducted four experiments: The first experiment aimed to evaluate the effect of kaolin in individual combinations with sucrose and glycerol and drying to two levels of water activity (0.3 and 0.2) on the viability of submerged *Batkoa* propagules. In the second experiment, we encapsulated the submerged *Batkoa* propagules in different concentrations of kaolin and assessed their viability before and after drying in a fluidized bed (Mini/Midi, Glatt Group^®^, Binzen, Germany), following the drying conditions described in Section 2.4.2. The third experiment evaluated the shelf life of the encapsulated formulations with or without kaolin that were washed with water or sucrose 4% over 45 days at 4 °C and 26 °C. Finally, in the fourth experiment, we assessed the sporulation potential of the hydrated beads kept on water agar for 30 days.

#### 2.4.1. Combination of Osmoprotectants with Kaolin

Submerged propagules, produced as described in Section 2.2, were separated from the liquid medium by filtration through three layers of gauze to retain the mycelia. The remaining liquid was centrifuged at 5000 rpm for 5 min, the supernatant was discarded, and the pellet was resuspended in one of the following solutions: distilled water, 5% kaolin, 4% sucrose, 4% sucrose + 1% kaolin, 4% sucrose + 5% kaolin, 4% glycerol, 4% glycerol + 1% kaolin, or 4% glycerol + 5% kaolin. The pH was adjusted to 6.7 to resemble the fermentation on the last day. Each formulation was then filtered through filter paper (6–8 μm pore size, Whatman^®^, Maidstone, United Kingdom CAT n° 1001-076) using a vacuum pump coupled to a Büchner funnel. The samples were subsequently conditioned, according to the methodology described by Iwanicki et al. [14], to slow drying over 15–18 h in a laminar flow chamber at a relative humidity of 50–60% until the water activity (a_W_) reached 0.3. They were then dried with silica gel to reduce the water activity to 0.2 [13,16,29]. Viability was analyzed before drying, after slow drying, and after silica drying. The entire experiment was conducted three times using different fungal preparations.

#### 2.4.2. Encapsulation with Different Kaolin Concentrations

Submerged propagules, produced as described in Section 2.2, were pooled from six flasks and centrifuged at 3500 rpm and 18 °C for 15 min. The resulting propagule material was washed twice in Phosphate-Buffered Saline (PBS), maintaining the original volume, and subjected to repeated centrifugation under the same conditions. After the final centrifugation step, the supernatant was discarded, and the pelletized propagule material was used for encapsulation.

To determine the optimal kaolin concentration, an experiment was conducted testing five kaolin concentrations (0%, 2%, 4%, 8% and 10%). Before encapsulation, sodium alginate (Manugel GMB, FMC Corporation, Philadelphia, PA, USA, lot No. G7708901) was dissolved in ultrapure water to a final concentration of 4% and autoclaved for 6 min at 121 °C. The encapsulation suspension was prepared by mixing heat-sterilized kaolin with sodium alginate according to the treatment concentrations. Submerged propagules, standardized at 1 ×10^7^ propagules mL^−1^, were added to achieve a final concentration of 10%. The suspension was gently shaken with the aid of a magnetic stirrer for 5 min. For bead formation, the solution was dropped into a stirred solution (350 rpm) of calcium chloride (0.1 M) (Carl Roth GmbH + Co, Karlsruhe, Germany, batch No. 5239.3) using a syringe (BD PlastipakTM, 20 mL, Bergen, New Jersey, USA) fitted with a needle (diameter 0.90 × 40 mm, Sterican, B. Braun AG, Melsungen, Germany). Each bead had an average weight of 0.0172 g for the control treatment and 0.0214 g for the Kaolin 10% treatment, with a final concentration per bead ranging from 1.72 to 2.14 × 10^5^ submerged propagules mL^−1^. The beads were kept in the solution under agitation for 20 min for the crosslink reaction and then washed with ultrapure water or sucrose for 20 min [22]. 

A fluidized bed (Mini/Midi, Glatt Group^®^, Binzen, Germany) was used to dry the beads in batches. Each batch comprised 150 g of moist beads dried with a constant air volume of 566 m^3^/h at a steady inlet air temperature of 40 °C. Throughout the process, inlet and outlet air temperature and relative humidity were recorded with a data logger (635, Testo SE & Co. KGaA^®^, Lenzkirch, Germany). The beads were dried until they reached a water activity below 0.2, measured with a water activity meter (LabMaster, Novasina, Lachen, Switzerland) at 25 °C. Pre-experiments were conducted to determine the drying time necessary to reach these water activities at 40 °C. The predetermined drying time values are shown in Table 1. The diameter of the beads was determined using a digital microscope (Zeiss^®^ Axiocam 208 color, Oberkochen, Germany) and analyzed with the Zen 3.0 Blue edition software. Each bead’s diameter was measured in four different cross-sections, following the methodology outlined by Vemmer [30]. The entire experiment was repeated twice.

### 2.5. Viable Propagules per Bead (VPB) and Sporulation

The viability of submerged propagules mixed with kaolin and osmoprotectants was evaluated by determining the viable propagules per bead concentration. The quality of beads formed through encapsulation and subjected to drying in the fluidized bed was assessed to determine the concentration of viable propagules per bead and sporulation. For the VPB determination, 15 beads of each treatment (fresh and dry) were dissolved in 10 mL of a solution adapted from Mater et al. [31] containing 0.03 M citric acid and 0.05 M sodium carbonate, with pH adjusted to 6.8 for 40 min on a rotary shaker at 230 rpm and 28 °C. The number of viable propagules was determined by counting colonies grown at 28 °C after 48 h on PDA. 

Sporulation was recorded by placing five beads on PDA in a Petri dish (Ø = 70 mm) and incubating them at 28 °C for 15 days. The discharged conidia were harvested from the plates with 10 mL PBS (10%). The concentration of conidia was determined using the Neubauer chamber (hemocytometer) at 400× magnification (Zeiss^®^ Axiostan plus, Oberkochen, Germany).

### 2.6. Shelf Life

Based on the prior results, we selected the formulation of beads produced with submerged propagules and 10% kaolin to further evaluate viability during storage. In this experiment, we assess the effect of kaolin and the washing of beads before drying. We compare washing with water to cleaning with a sucrose solution (4%), as sucrose is known to act as a thermoprotector [32]. Beads were dried in a fluidized bed, as described in Section 2.4.2, and stored at 4 °C or 28 °C in individual vacuum-sealed aluminized packages (5.0 × 5.0 cm), with fifteen beads in each package. The production of primary conidia and viable propagules per bead by colony-forming unit was evaluated immediately after drying and after 7, 15, 30, and 45 days of storage. One package was evaluated for each temperature condition at each specified time point. The experiment was repeated twice using new fungal batches.

### 2.7. Sporulation of Beads over Time in a Humid Environment

In this experiment, we evaluated the sporulation of freshly prepared beads containing *Batkoa* submerged propagules and the viability of primary conidia formed on these beads. The beads were placed on water agar plates and incubated at 28 °C under a 12 h photoperiod for 30 days. Beads were prepared following the procedure outlined in Section 2.4, employing the same treatments as those determined for the shelf-life study: Control + H_2_O, Control + Sucrose, Kaolin + H_2_O, and Kaolin + Sucrose.

After the beads were formed in the CaCl_2_, the solution was discarded using a mesh, and water or sucrose was added to the same container where the beads were produced and incubated for 20 min. Subsequently, the content was discarded, and the beads were separated. The viability of the primary conidia was assessed by plating 100 μL of the suspension onto potato dextrose agar (PDA) (6 cm in diameter, Rodac™ type, Dickinson and Co., Franklin Lakes, NJ, USA) and incubating it at 28 °C for 8 h. Viability was assessed by randomly counting 100 conidia and distinguishing between germinated and non-germinated cells. Conidia with germ tubes equal to or longer than half the length of the conidial cell were classified as germinated (viable). The viability percentage was calculated using the following formula: viability = number of germinated cells/total number of cells counted per sample. Sporulation was performed according to the procedure outlined in Section 2.5. To observe conidia release from the beads, Rodac plates containing five beads from each treatment were placed in the Reshape Microbiology Platform (Reshape Biotech^®^, Copenhagen, Denmark). This platform was programmed to capture images every 10 min throughout the experiment, enabling a detailed observation of sporulation dynamics over time. The experiment was replicated twice using new fungal batches. Treatments were analyzed after 2, 7, 12, 17, 22, 27, and 30 days. Each time, beads from three Petri dishes per treatment were analyzed.

### 2.8. Statistical Analysis

Bioassays with insects and formulation experiments were conducted using a completely randomized design, repeated thrice. Survival data were visualized using Kaplan–Meier estimates. The survival distribution curves were compared using the log-rank test followed by the Holm–Sidak multiple comparison procedure at a 5% significance level. The effect of combining kaolin with osmoprotectant treatments across different water activities and within the same treatment on viability was assessed using a paired *t*-test or Wilcoxon test at *p* < 0.05. Within the same water activity, treatment effects were analyzed using the multiple pairwise Tukey’s HSD test at *p* < 0.05 or the Kruskal–Wallis test at *p* < 0.05 when normality and homogeneity of variance were not met. Colony-forming units and sporulation data were log10-transformed before the analysis of variance to meet normality assumptions. For the longitudinal dataset on the shelf life of *Batkoa* beads, measured by sporulation and viable propagules per bead over time (days), we fit a generalized linear mixed model (glmer) with treatments, storage condition, and time as fixed effects, while sampling packages and bioassays (experimental repetitions) were scored as random effects. Treatment means were compared using Tukey’s HSD post hoc test at *p* < 0.05 for each evaluation day. The sporulation data of fresh beads incubated on PDA plates over time were fitted to a generalized linear model (GLM) with a negative binomial distribution, and estimated marginal means with 95% confidence intervals were used to assess treatment significance. All analyses were performed using the free statistical software R version 4.4.2 [33], and plots were built on SigmaPlot^®^ version 14.0 (Systat Software, Inc., Chicago, IL, USA)

## 3. Results

### 3.1. Bioassay with Dalbulus maidis

**Figure 1:**
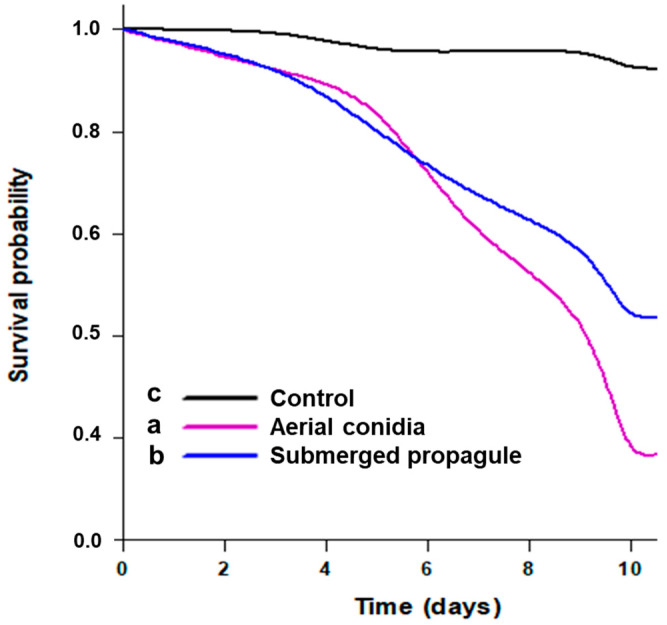
Survival probability of leafhopper *Dalbulus maidis* adults after exposure to primary conidia and submerged propagules of *Batkoa* sp. ESALQ1199 over time. Different letters indicate significant differences between the groups according to a log-rank test (*p* < 0.05).

In this experiment, we evaluated the infectivity and virulence of *Batkoa* primary conidia and submerged propagules against corn leafhopper adults, assessing the median survival time (MST) to determine the time taken by the fungus to kill 50% of insects and mycosis to determine insect sporulation rate. Our results demonstrated that the primary conidia and submerged propagules successfully killed 82.4% and 57.8% of corn leafhopper adults, respectively, while the control treatment showed only 7.9% mortality. Insects displayed signs of mycosis by *Batkoa*, with mycosis rates of 46% for primary conidia and 31% for submerged propagules. Notably, this is the first documented instance of *Batkoa* sp. submerged propagules exhibiting infectivity against an insect. The survival curves of leafhoppers’ exposure to primary conidia and submerged propagules indicated a significant difference in lifespan, with statistical differences observed between the groups (χ^2^ = 373.48; df = 2; *p* < 0.001) (Figure 1). Untreated adults had a higher survival time than those treated with either primary conidia or submerged propagules, confirming the efficacy of the fungal treatments. Among the treatments, primary conidia had the lowest MST of 8 days, indicating the highest lethality, followed by submerged propagules with an MST of 9 days.

### 3.2. Formulation Experiments

#### 3.2.1. Combination of Osmoprotectants with Kaolin

In this experiment, the effects of combining kaolin with the osmoprotectants sucrose and glycerol at two concentrations, 1% and 5%, as well as the drying time to achieve two water activities, 0.2 and 0.3, on the viability of submerged *Batkoa* sp. propagules were evaluated. The aim was to select the best combination of co-formulants for the subsequent encapsulation experiment of the submerged propagules. Before the drying process, the initial viability of the propagules was assessed after mixing with the co-formulants, resulting in values ranging from 96% to 100%.

**Figure 2:**
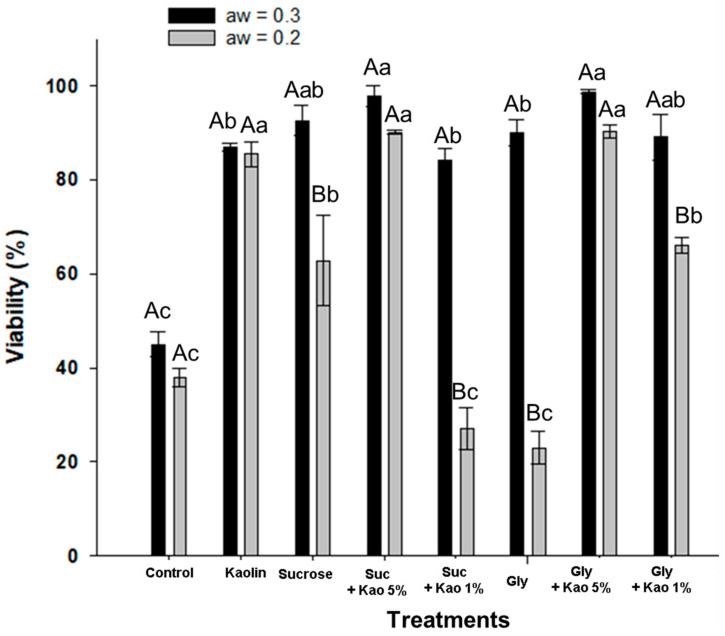
Viability of *Batkoa* sp. ESALQ 1199 submerged propagules dried with different co-formulants, individually or in combination (kaolin [kao], sucrose [suc], and glycerol [gly] at 1% and 5%). Initially, water activity was reduced to 0.3 ± in a drying chamber, followed by a further decrease to 0.2 ± by mixing the propagules with carriers in silica. Means (±SE) followed by different uppercase letters indicate statistical differences within the same treatment across different water activities, as determined by the Wilcoxon test or paired *t*-test (*p* < 0.05). Means (±SE) followed by different lowercase letters indicate statistical differences among treatments within the same water activity, as determined by the Kruskal–Wallis test or multiple pairwise Tukey’s HSD (*p* < 0.05).

The concentration of viable propagules per treatment was significantly affected by the final water activity (*p* < 0.001) and the different co-formulants (*p* < 0.001), varying from 23 ± 7.82% for the treatment with only glycerol mixture dried to a_W_ = 0.223 ± 0.03 to 98.81 ± 1.19% for the treatment with 5% kaolin mixed with glycerol dried to a_W_ = 0.354 ± 0.025 (Figure 2). The mixture with co-formulants preserved the initial viability of the submerged propagules when dried to a_W_ = 0.322 ± 0.01, maintaining viability above 80% for all treatments. In contrast, the viability in the control treatment after drying to a_W_ = 0.321 ± 0.03, without co-formulant addition, was reduced to 45.00 ± 5.97%. These results indicate the sensitivity of submerged propagules to the drying process and highlight the importance of formulation in preserving fungal viability. While drying to a_W_ = 0.312 ± 0.01 was beneficial for the fungus, further drying in silica to a_W_ =0.201 ± 0.02 resulted in a significant reduction in propagule viability for the treatments with only sucrose, glycerol with 1% kaolin, sucrose with 1% kaolin, and glycerol. The viability of the latter two treatments dropped more than 50% compared to the same treatments dried to a_W_ =0.3, resulting in values lower than those observed in the control treatment. On the other hand, additional drying did not affect the viability of the submerged propagules in the control treatment, or the treatments with only kaolin, sucrose with 5% kaolin, or glycerol with 5% kaolin. Based on these results, kaolin and sucrose were selected as formulation additives for the next experiments.

#### 3.2.2. Selection of Kaolin Concentration

In this study, we investigated the effect of varying kaolin concentrations on the concentrations of viable propagules per bead (VPB) by observing the colony-formation method and the sporulation of beads composed of submerged propagules of the fungus *Batkoa*. We hypothesized that higher kaolin concentrations would reduce the pressure exerted during the drying process, as the kaolin acts as a filler, mitigating the impact on fungal propagules and preserving their viability.

**Figure 3:**
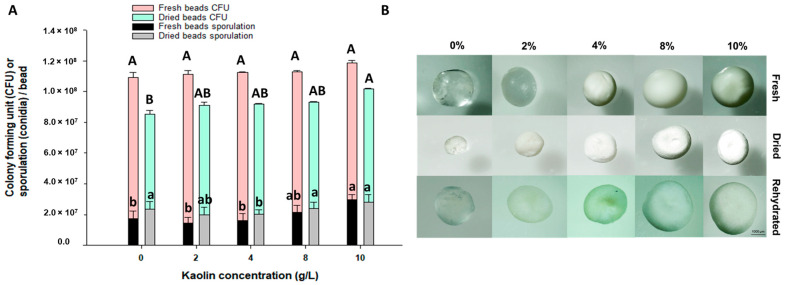
Colony-forming units and sporulation of Batkoa sp. ESALQ 1199 per bead encapsulated with five different concentrations of kaolin (0, 2%, 4%, 8%, and 10%) before and after drying in a fluidized bed dryer at 40 °C inlet. Means (±SE) followed by different letters between the same color bar indicate significant differences according to Tukey’s HSD test (*p* < 0.05) (**A**). Beads formed with varying concentrations of kaolin (0%, 2%, 4%, 8%, and 10%) in fresh, dry, and hydrated states (**B**).

Samples were analyzed in their fresh state and after drying in a fluidized bed at 40 °C. Overall, variations in kaolin concentration had minimal influence on VPBs and sporulation. However, a decrease in VPB was observed post-drying for all treatments (Figure 3A,B). Kaolin concentration only affected the formation of VPBs in dried beads, with beads containing 10% kaolin exhibiting a higher VPB compared to those without kaolin (*p* < 0.01). Regarding sporulation, we found that the drying process had also minimal effect on the fungus’s ability to sporulate (*p* < 0.01). The presence of kaolin did not enhance the sporulation process, as the treatment without kaolin sporulated at the same rate as treatments with 2%, 8%, and 10% kaolin. These data suggest that higher kaolin concentrations do not significantly influence *Batkoa* sp. VPB in fresh beads. 

The average diameter of the beads varied significantly depending on the kaolin concentration (Table 2). Among the fresh beads, the control treatment, represented as 0% kaolin, had the smallest diameter at 1.95 ± 0.03 mm, while the largest diameter was observed in the 4% kaolin treatment at 2.37 ± 0.04 mm. Upon drying, it was observed that the bead diameter was larger with higher kaolin concentrations. Beads formed with 8% and 10% kaolin had higher diameters of 1.45 ± 0.02 mm and 1.58 ± 0.02 mm, respectively. After hydration, the diameters of the beads did not match those observed for the fresh beads; however, they followed the trend observed for the dry beads: the higher the kaolin concentration, the larger the diameter, except for the 8% and 10% concentrations, where the diameter was the same.

### 3.3. Shelf Life

Based on the aforementioned results, we selected the formulation of beads produced with submerged propagules and 10% kaolin to further evaluate viability during storage. In this experiment, we also evaluated the effect of washing the beads before drying in a sucrose solution (4%), as sucrose is known to act as an osmoprotectant, using water as a washing detergent as the control.

**Figure 4:**
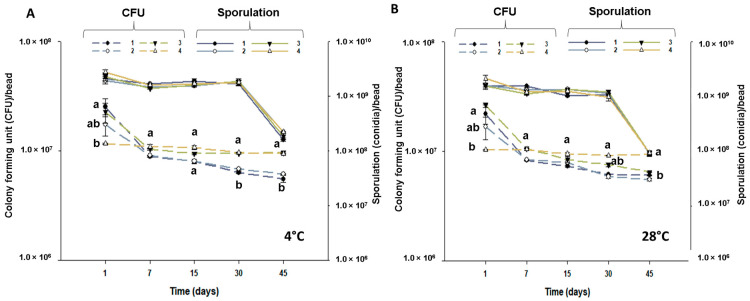
Colony-forming units and sporulation of *Batkoa* sp. ESALQ 1199 per bead prepared without kaolin and washed with 4% sucrose (lines numbered 1), without kaolin and washed with water (lines numbered 2), with kaolin 10% and washed with water (lines numbered 3), and with kaolin 10% and washed with 4% sucrose (lines numbered 4). Beads were dried in a fluidized bed at an inlet temperature of 40 °C and stored at 4 °C (**A**) and 28 °C (**B**). Different letters at the same time indicate the statistical differences between treatments for colony-forming units according to Tukey’s HSD test (*p* < 0.05). There is no statistical difference between treatments carried out at the same time for variable sporulation, regardless of storage condition.

Concentrations of viable propagules per bead (VPB) by colony-forming unit were significantly affected by storage conditions (*F* = 2.03; df = 1; *p* < 0.001) and the interaction between treatment and time (*F* = 8.47; df = 3; *p* < 0.001). Desiccation tolerance, as indicated by the VPB data from the first day, revealed lower values for the treatment with 10% kaolin followed by a 4% sucrose wash (line 4) compared to the treatment without kaolin followed by a 4% sucrose wash (line 1) and the treatment with 10% kaolin followed by washing in water (Figure 4). However, over time, the treatment with 10% kaolin and a 4% sucrose washing step was the only one that remained stable, showing no decline in VPB values under either storage condition. The most significant decrease in VPB occurred after seven days of storage. In general, beads prepared with kaolin showed higher VPB over time than those without kaolin.

Sporulation was significantly affected by storage condition (*F* = 37.51; df = 1; *p* < 0.001) and time (*F* = 214.60; df = 3; *p* < 0.001), but not by the treatments (*F* = 0.66; df = 3; *p* = 0.574). Beads made with or without kaolin and washed or unwashed with sucrose solution showed comparable sporulation values on each evaluated date. In contrast with the VPB observations that showed a sharp decrease after seven days for most treatments, the highest decrease in sporulation was observed between days 30 and 45, regardless of the storage condition (Figure 4).

#### Sporulation of Fresh Beads over Time

**Figure 5:**
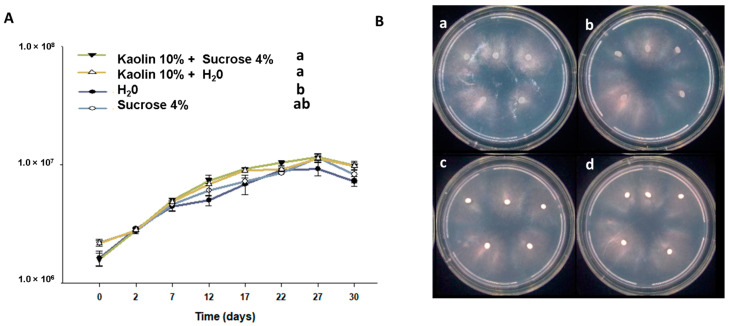
Duration and intensity of conidial discharge by *Batkoa* sp. from different beads within the first 30 days of bead preparation with incubation at 28 °C and a 12 h photophase. Different letters in the legend indicate significant differences according to estimated marginal means (**A**). Sporulated beads after fifteen days incubated at 28 °C. Beads prepared without kaolin and washed with water (**a**), without kaolin and washed with 4% sucrose (**b**), with kaolin 10% and washed with water (**c**), and with kaolin 10% and washed with 4% sucrose (**d**) (**B**).

Sporulation of *Batkoa* after encapsulation in calcium alginate beads was significantly influenced by both treatments (*F* = 7.48; df = 3; *p* < 0.001) and storage duration (*F* = 740.61; df = 7; *p* < 0.001). Sporulation increased throughout the incubation period (Figure 5), reaching a plateau after 27 days, with a maximum average production of 11.4 ± 0.16 × 10^6^ conidia per bead for the treatment with kaolin washed with water. Washing the kaolin beads with 4% sucrose significantly increased the total number of conidia discharged (11.6 ± 0.28 × 10^6^) compared to beads without kaolin washed with water (9.25 ± 0.50 × 10^6^), though the results did not significantly differ from the treatment without kaolin washed with 4% sucrose (11.5 ± 0.37 × 10^6^). The viability of conidia produced on all dates exceeded 95% for all formulations, and after 30 days of incubation, sporulation decreased across all treatments.

## 4. Discussion

Optimizing formulations for *Batkoa* sp. has presented a significant challenge to researchers. While promising formulations have been developed for a few other entomophthoralean species, such as *Pandora neoaphidis* [34], *Pandora nouryi* [35,36], *Pandora cacopsyllae* [22], and *Conidiobolus obscurus* [37], there is a notable absence of studies focused on formulation development for the genus *Batkoa*. Therefore, in the present study, we developed formulations via ionic gelation by incorporating submerged propagules of *Batkoa* sp. (ESALQ1199) in calcium alginate with kaolin. These formulations demonstrated a high capacity for sporulation following desiccation and shelf life for at least 30 days at both 26 °C and 4 °C. Although sporulation decreased over time, a sporulation rate of 10⁷ conidia per bead was still observed after 45 days. However, since the shelf life was not evaluated beyond 45 days, it is unclear whether sporulation would decline further. Considering that *Batkoa* conidia are larger than those of ascomycetous fungi and typically require fewer conidia to achieve insect mortality, this quantity could be sufficient to cause significant mortality in leafhoppers under field conditions. Also, this fungus could be used as an inoculative release, considering its ability to cause epizootics naturally. However, further studies are necessary to confirm these hypotheses. 

Our findings indicate that the tendency for sporulation to increase with higher kaolin concentrations was more pronounced in the desiccated beads, suggesting that kaolin may play a protective role during the drying process and or a stimulatory role in enhancing sporulation afterward. The main component of kaolin is aluminum silicate kaolinite, which has oxygen-scavenging activity [38] and could be beneficial for fungi during storage. Furthermore, kaolin is commonly used in various bio-based commercial formulations, including wettable powders and dispersible granules. These results suggest that kaolin is a compatible and effective inert substance in *Batkoa* formulations. Additionally, we examined whether washing the beads with a 4% sucrose solution, intended for thermoprotection, could enhance desiccation tolerance and preserve viability over time. Our results showed that sucrose washing did not significantly improve desiccation tolerance, viability, or sporulation (conidial production) during storage.

In contrast, Muskat et al. [22], demonstrated that encapsulating *Pandora cacopsyllae*-submerged hyphal material in calcium alginate beads enriched with various fillers, such as starch, chitin, and skimmed milk, led to a substantial increase in conidia discharge of approximately 9 × 10^6^ conidia per bead, mainly when skimmed milk was included in the formulation. Adding these nutrients appeared to create a more advantageous environment for conidial production. Similarly to Muskat et al. [22], Shah et al. [34] reported a significant increase in the discharge of *Pandora neoaphidis* conidia when the fungus was encapsulated in alginate beads that included sucrose, potato starch, or chitin. They measured the conidia discharge area, finding increases of approximately 89.7 conidia mm^−2^ with the addition of 2% sucrose, 17.7 conidia mm^−2^ with 5% starch, and 25.2 conidia mm^−2^ with 5% chitin. These findings suggest that certain nutrients in a formulation can significantly boost conidial output. In a related study, Zhou and Feng [36] developed alginate beads containing millet powder and *Pandora nouryi* mycelia. They observed that these beads sporulated at approximately 28.4 × 10^4^ conidia per bead, nearly double the rate observed in treatments that did not include millet powder. In our approach, we did not incorporate any additional nutrients into the bead preparation besides washing it with sucrose solution, which might have otherwise enhanced the sporulation reported in this study. Despite the variation in methodologies, fungal species, and nutrient compositions across these studies, the collective findings highlight the critical influence of nutrient additives on the efficacy of alginate bead formulations for fungal encapsulation and subsequent conidial production. 

The drying process of formulations is essential for reducing the metabolic rate of microorganisms [39,40] by decreasing the water content [41] and extending shelf life. In this study, we demonstrated that submerged propagules, formulated with kaolin alone or combined with sucrose or glycerol, preserved their viability when subjected to additional drying to achieve a water activity level of 0.2. Li et al. [42] used a simple drying method (similar to air drying), adapted from a patent by McCabe and Soper [43], with a ventilated chamber at 4 °C; after filtering the *Zoophthora radicans* and *Pandora neoaphidis* (formerly *Erynia neoaphidis*) propagules, the biomass was kept overnight in a laminar flow at 4 °C and high relative humidity. After the drying process, the material was stored at 4° and −20 °C. The authors achieved relatively high viability, around 55–50%, after 80 days of storage at 4 °C. Similarly, Pell et al. [44] dried mycelia produced by *Z. radicans* treated with maltose, storing it at a temperature of 4 °C. The authors obtained 50% viability after 80 days of storage. Using other materials as desiccants and oxygen scavengers, Leite et al. [45] determined the effects of combinations of silica and glycerol (as a desiccant factor), vacuum, and Ageless^®^ ZPT-200 (as an oxygen scavenger) on *Batkoa* and *Furia* mycelia shelf life. They concluded that the combination of silica and Ageless^®^ resulted in 86% and 100% viability for *Batkoa* and *Furia*, respectively, after 90 days of storage at 3 ° or 23 °C. 

The formulation developed for *Batkoa* sp. (ESALQ1199) is designed for field application in a granular form, targeting the area around the corn ear, where adult corn leafhoppers are commonly found, or at the base of sugarcane clumps, aiming to control the sugarcane spittlebug *M. fimbriolata*. In both environments, the microclimatic conditions might be similar, characterized by high temperature and humidity. Our sporulation studies under controlled conditions of high humidity and temperature (28 °C) indicate that kaolin-enriched fresh beads can remain sporulating for extended periods, reaching a peak after 27 days, with the produced conidia demonstrating high viability. Similar studies by Muskat et al. [22] show potential sporulation for up to 12 days for the fungus *Pandora cacopsyllae*, and also observed sporulation levels of 9.57 × 10^6^ conidia per bead. Olsen et al. [46], through statistical analysis, estimated a shorter sporulation period for *P. cacopsylae*, lasting up to 5 days, with a peak around 2 days for unformulated mycelial mats. Our *Batkoa* beads demonstrated a sporulation rate of 1.0 × 10^7^ conidia per bead, similar or even higher than that reported in other studies. However, this sporulation rate may be influenced by factors such as the composition of the formulation, the amount of active ingredient per bead, and the intrinsic characteristics of each species and isolate, which limits the direct comparison of the results between the studies.

This study is the first to highlight the infectivity of submerged propagules of *Batkoa* sp. (ESALQ1199) against adult corn leafhoppers (*Dalbulus maidis*). In nature, entomophthoralean species typically infect their hosts through primary conidia ejected from previously infected cadavers [47]. When these primary conidia get in contact with potential hosts, they rapidly germinate, infecting their host and leading to the insect’s death. This passive form of control is highly effective, given that entomophthoralean fungi require only a few conidia to infect a host (usually fewer than 10) [48], and can be dispersed across the environment, reaching considerable distances. In our bioassay, we employed an approach simulating inundative control, wherein the infective propagules are sprayed in an aqueous suspension at high concentrations (typically around 1 × 10^12^ conidia/ha) directly onto the host insect. This approach is commonly used for hypocrealean fungi, such as *M. anisopliae* and *Beauveria bassiana*, to manage agricultural pests in crop fields. In this study, control of the corn leafhopper was achieved by applying non-formulated submerged propagules and conidia. The shortest mean survival time of 50% of individuals was observed 7 days after treatment with the primary conidia, whereas the submerged propagule treatment resulted in an approximate survival time of 9 days. This is much longer than other reports on the same genera, *Batkoa*. Hajek and Harris [49] determined for *Galleria mellonella* infected with *Batkoa major* a lethal time TL_50_ of 4 days after infection. In contrast, Lopes et al. [50] reported an overall mortality rate of 35% for adult *D. maidis* after seven days of exposure to conidia from the genera *Beauveria*, *Metarhizium*, and *Cordyceps*. Additionally, Muskat et al. [51] reported that an isolate of *Pandora cacopsyllae* demonstrated a rapid speed of kill, with a TL_50_ (time to 50% mortality) occurring within 5 to 6 days for *Cacopsylla picta* and *C. pyri*. Similarly, Görg et al. [52] found comparable results when evaluating the virulence of *P. cacopsyllae* on different psyllid hosts, with a median survival time of approximately 5 days for both *C. picta* and *C. pyri*.

Considering the inundative biological control strategy, i.e., the spraying of high concentrations of submerged propagules of *Batkoa* sp., and the higher virulence of entomophthoralean fungal conidia compared to hypocrealean entomopathogenic fungi, we anticipated an even higher mortality rate over a short period than what was observed in this study. These results may be attributed to the characteristics of the sprayed propagules. We hypothesize that the submerged propagules obtained in this study from liquid culture are secondary conidia. Thus, additional time may be required for these spores to germinate, develop hyphae, which can produce new primary conidia capable of infecting the corn leafhopper. The data presented herein clearly demonstrate the viability of an encapsulated formulation of submerged propagules of *Batkoa* sp. ESALQ1199 with kaolin, utilizing a set of optimized parameters in a fluidized bed dryer. Furthermore, the submerged propagules produced in liquid media exhibited resilience during the drying stabilization process. Additional studies are required to characterize the submerged propagules of *Batkoa* sp. 

## 5. Conclusions

The application of submerged propagules of *Batkoa* sp. (ESALQ1199) proved effective in controlling populations of adult corn leafhoppers (*D. maidis*), demonstrating similar virulence compared to primary conidia. We successfully developed an encapsulated formulation of submerged *Batkoa* sp. propagules that retained the same concentration of viable propagules per bead and the number of conidia produced (sporulation) for 30 days at 28 °C. These findings underscore the importance of optimizing formulations to develop an effective, eco-friendly microbial insecticide based on submerged propagules of *Batkoa* sp. to specifically target corn leafhoppers.

## Figures and Tables

**Table 1:** Technical drying parameters for beads from four treatments using the isolate *Batkoa* ESALQ 1199. Each batch containing 150 g of moist beads was dried using a consistent air volume of 566 m^3^/h at a constant inlet air temperature of 40 °C.

Treatment	Inlet (°C)	Outlet First (°C)	Outlet Final (°C)	RH% of the Air–Start	RH% of the Air–Final	Water Activity	Water Content (%)	Drying Time
Control + H_2_O	40	24.80	38.20	20.80	1.30	0.14	94.31	90 min
Control + Sucrose 4%	40	32.80	37.20	10.40	1.30	0.21	93.42	180 min
Kaolin 10% + H_2_O	40	28.50	31.60	17.30	1.40	0.07	97.27	90 min
Kaolin 10% + Sucrose 4%	40	31.30	36.50	16.40	1.30	0.21	95.39	180 min

**Table 2:** Diameter of *Batkoa* ESALQ 1199 beads prepared with different concentrations of kaolin (0%, 2%, 4%, 8%, and 10%) after drying in a fluidized bed. Means (±SE) followed by different letters within each drying time (column) indicate significant differences according to Tukey’s HSD test (*p* < 0.05).

Kaolin Concentration (%)	Fresh (mm)	Dry (mm)	Rehydrated (mm)
0	1.95 ± 0.03 c	0.83 ± 0.02 e	1.23 ± 0.04 c
2	2.09 ± 0.05 bc	1.14 ± 0.01 d	1.50 ± 0.03 b
4	2.37 ± 0.03 a	1.22 ± 0.01 c	1.56 ± 0.05 b
8	2.14 ± 0.05 b	1.45 ± 0.01 b	1.97 ± 0.03 a
10	2.10 ± 0.04 b	1.58 ± 0.02 a	1.97 ± 0.04 a

## Data Availability

Not applicable.
